# Metapopulation Dynamics Enable Persistence of Influenza A, Including A/H5N1, in Poultry

**DOI:** 10.1371/journal.pone.0080091

**Published:** 2013-12-02

**Authors:** Parviez Rana Hosseini, Trevon Fuller, Ryan Harrigan, Delong Zhao, Carmen Sofia Arriola, Armandoe Gonzalez, Matthew Joshua Miller, Xiangming Xiao, Tom B. Smith, Jamie Holland Jones, Peter Daszak

**Affiliations:** 1 EcoHealth Alliance, New York, New York, United States of America; 2 Center for Tropical Research, Institute of the Environment, University of California Los Angeles, Los Angeles, California, United States of America; 3 Department of Ecology and Evolutionary Biology, University of California Los Angeles, Los Angeles, California, United States of America; 4 Department of Botany and Microbiology, Center for Spatial Analysis, University of Oklahoma, Norman, Oklahoma, United States of America; 5 Laboratory of Preventive Veterinary Medicine, School of Veterinary Medicine, San Marcos Major National University, Lima, Peru; 6 Smithsonian Tropical Research Institute, Panamá, República de Panamá; 7 Woods Institute for the Environment and Department of Anthropology, Stanford University, Stanford, California, United States of America; The Pirbright Institute, United Kingdom

## Abstract

Highly pathogenic influenza A/H5N1 has persistently but sporadically caused human illness and death since 1997. Yet it is still unclear how this pathogen is able to persist globally. While wild birds seem to be a genetic reservoir for influenza A, they do not seem to be the main source of human illness. Here, we highlight the role that domestic poultry may play in maintaining A/H5N1 globally, using theoretical models of spatial population structure in poultry populations. We find that a metapopulation of moderately sized poultry flocks can sustain the pathogen in a finite poultry population for over two years. Our results suggest that it is possible that moderately intensive backyard farms could sustain the pathogen indefinitely in real systems. This fits a pattern that has been observed from many empirical systems. Rather than just employing standard culling procedures to control the disease, our model suggests ways that poultry production systems may be modified.

## Introduction

Highly pathogenic influenza A/H5N1, colloquially referred to as “Avian Flu”, was first identified in Hong Kong in 1997 as a cause of fatal respiratory illness in humans [1]. After spreading from Asia to Europe and Africa in 2005, it has persisted since then. It has been reported from wild waterfowl in Asia [2] and Europe [3]–[5], as the cause of outbreaks of poultry disease in Asia, Europe and Africa [6]–[12], and as the cause of repeated zoonotic transmission to humans in Asia, Europe and Africa [13]–[16]. Although human-to-human transmission is rare, this strain of avian flu represents a significant pandemic threat, with 360 reported human deaths by the end of 2012 [17], and we now know artificial selection has been able to create a strain of A/H5N1, that has only a modicum of mutations from naturally occurring strains, that can make the virus transmissible among mammals while remaining pathogenic [18]. Yet it remains unclear why this has not occurred outside the laboratory. In addition, the case fatality rates for human A/H5N1 infections remain high [17], as do the mortality rates in chickens, some breeds of domestic ducks, and some species of wild birds [19], making control measures difficult and urgent [20]. Although many agencies and governments have tried to control A/H5N1 spread with some degree of success, it has not been eradicated nor displaced by a less pathogenic strain of Influenza [21], [22].

Indonesia, Egypt and Vietnam represent the three main foci of human A/H5N1, with over 75% of human cases occurring there [17]. Twelve other countries have reported sporadic and occasional human cases and 48 additional countries have reported infected animals [17], [23] with no reported spillover to people. Thus a key question is how A/H5N1 has persisted across the Eastern Hemisphere, but with only very distinct, geographically disparate foci of human cases. One explanation for this may be differences in the nature of poultry farming in affected versus unaffected countries. In particular, a number of studies have proposed backyard poultry rearing as a key risk factor for human infection [8], [24]–[27]. However, many countries that have dense human populations, extensive backyard poultry rearing, and repeated presence of A/H5N1, have reported no or only a few human cases (e.g. Bangladesh, India) [17].

At a large spatial scale, the presence of A/H5N1 in Asia has been correlated with duck density and rice cropping patterns [21], [28], [29], and its spread has been linked to bird migration and global poultry trade [30]–[36]. However, because of high livestock and human mortality, and the short duration of outbreaks, A/H5N1 dynamics have been difficult to study. Here we examine the spatial dynamics of A/H5N1 using theoretical models of disease spread and attempt to understand its ability to persist in different types of domestic poultry rearing operations.

It is a well-established theory in ecology that spatial structuring of a population, as opposed to a single well-mixed population, may alter dynamics [37], [38]. Spatial structure can dampen cycles, allow for co-existence of pathogens with hosts, and generally enable the long-term persistence of often lethal pathogens within a host population [39]–[41]. Employing a metapopulation approach has been successfully used to understand the dynamics of measles in small cities and towns [42], the sporadic nature of Hendra virus outbreaks [43], rabies in wild and domestic dogs [44], and cattle diseases [45]. In the current paper, we apply this understanding specifically to the case of A/H5N1 in domestic ducks, and poultry more broadly. We will demonstrate that spatial structuring can allow A/H5N1 to persist for an indefinite period of time without re-introduction from wild bird populations. We will also investigate the effects of some possible control measures, such as culling and cleaning. While these measures may help reduce the impact and spillover risk of A/H5N1, they may not be able to eliminate unless the effort put into making them effective is enormous.

## Methods: Model

We designed our model to represent a single species in several patches (*x ∈ {0,…J]*}. The model assumes a network of patches, representing farms, markets, or traders, linked together. Our model is a stochastic simulation developed using the Gillespie algorithm [46], [47] because we are focused on variability in persistence, but we present the deterministic skeleton of the model for simplicity and clarity. Within a patch, the model uses standard SIR dynamics for each species, with the addition of the presence of a common environmental reservoir of infection [48]. Within each patch (x), the dynamics follow these equations:
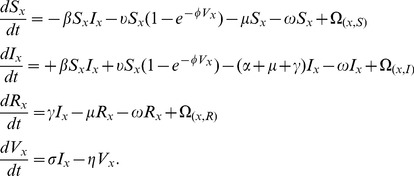
(1)


Where S represents the number of susceptibles, I infected, R recovered, and V virions in the environment. The initial conditions for all simulations are an entirely susceptible population, except for two infected individuals, located in two randomly chosen patches. We have included the indirect transmission model of Breban et al. [48], where each infected host sheds virus into the environment at rate (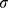), and virions in the environment degrade at rate (η). We use only the only the warm temperature parameter values (see Table 1), as we are focused here on poultry in subtropical and tropical environments. Transmission may occur indirectly from the environment 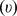, with a dose-dependence of likelihood of infection 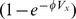, which depends on virion infectiousness (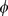). Direct transmission occurs at rate β_._ Infected animals recover at rate (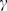), or die due to the disease at rate (α). In addition, there may be non-influenza related mortality (μ), for all categories (S_x_,I_x_,R_x_) of animals. We do not include birth, because for most real poultry systems, chicks are introduced by other means. Although not shown, the model keeps track of the number of dead animals from each infection category. For the introduction of a single infected individual into a fully susceptible population (S_0_), without any infectious virions in the environment, this model has a within-patch basic reproduction number of:
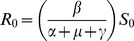



**Table 1:** Parameters of the model.

Symbol	Value/Range	Definition	Units	Reference
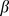	0.004	direct transmisison	animal^−1^ day^−1^	[73]
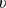	0–0.167	recovery rate	day^−1^	[51]–[53], [73]
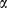	0–0.4	disease mortality rate	day^−1^	[51]–[53], [73]
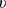	0.001	environmental uptake rate	day^−1^	[48]
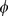	1.96 ⋅ 10^−4^	virion infectiousness	virion^−1^	Calculation from [48], ID_50_ from [74]
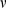	0.14	virus degradation rate	day^−1^	[48]
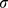	10^5^	shedding rate	day^−1^	[48]
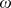	0.00–0.30	movement rate	day^−1^	[59]
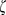	2	network neighborhood	patches	–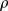	0.0596	re-wiring probability		–

Animals may leave the patch at rate (ω) from any class, presumably by human activity in the case of poultry. Animals that leave their current patch are then moved to another patch according to the function:
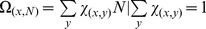
(2)


**Figure 1:**
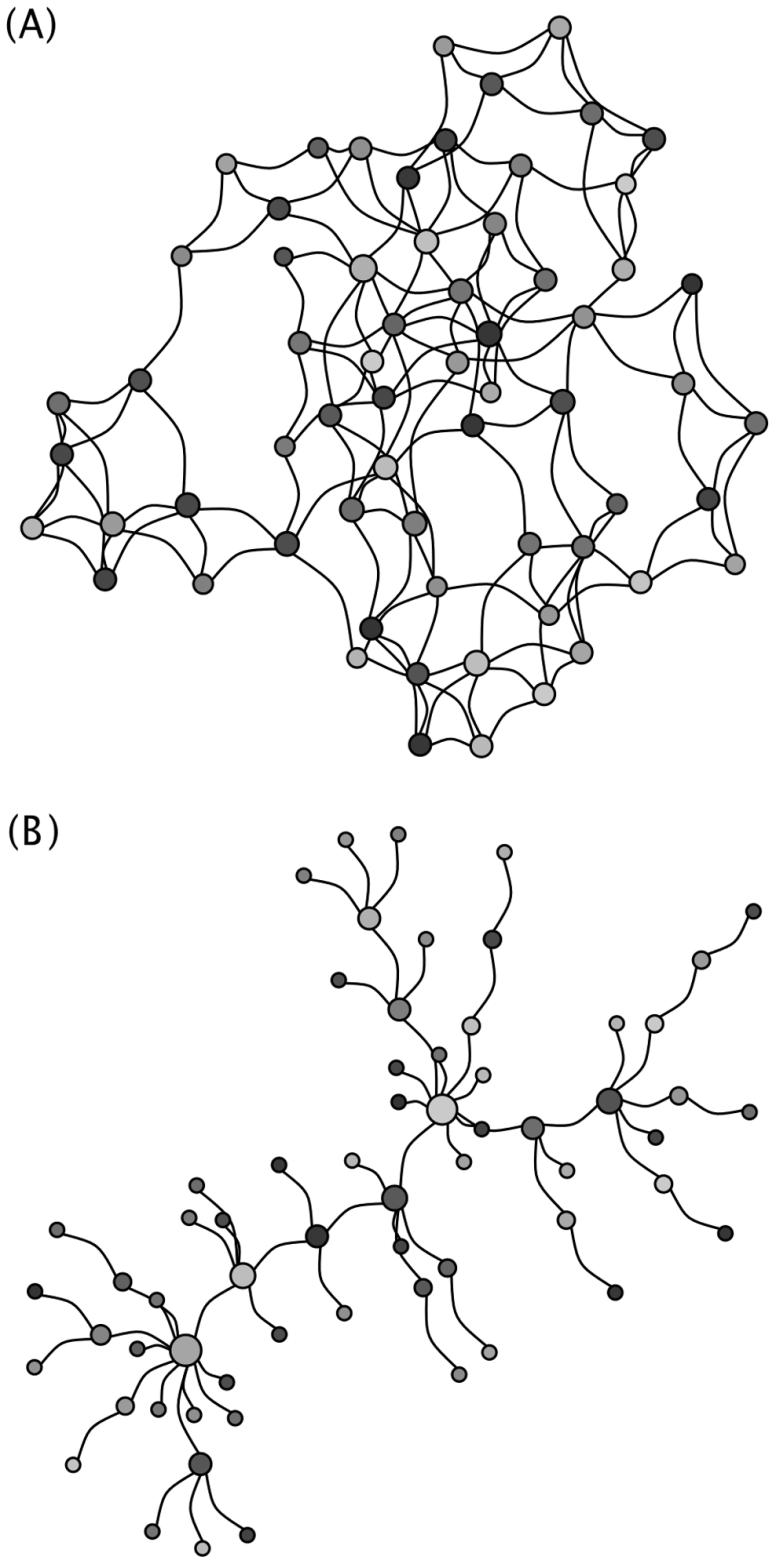
Two example random networks. (A) A Watt-Strogatz random small world network with 64 vertices, with 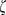 = 2.33 and ρ = 0.0596 (as in Table 1), (B) A Barabasi random network with 64 vertices, with the power of the preferential attachment set to 0.5 and the zero appeal to 1.0, both represent a network of 64 farms and markets, each with 500 animals.

Where χ_(x,y)_ represents the probability of movement between patches x and y, given a departure from patch y, and N represents the appropriate population (susceptible, infected or recovered), with the constraint that all χ_(x,y)_ must sum to 1 across all possible source patches. Here we use the row normalized adjacency matrix of a network model to specify the interconnectedness of the system. In the absence of relevant empirical data, we have examined two classic random networks. We have used the Watts-Strogatz random network model, known as the small-world model with neighborhood 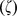 and re-wiring probability for global connections 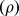 (e.g., Figure 1a) [49]. In order to examine the impact of network structure, we have also used a Barabasi random network (e.g., Figure 1b) [50].

Additionally, we have implemented a model of passive surveillance based control in the model. If the number of dead birds within a given period (τ_Crit_) exceeds a threshold (I_Crit_), it is presumed an authority would be notified given a probability of reporting (π_Report_), and that authority would test the living animals for infection, with a given probability of detection (π_Detect_). If the infection is detected, then all animals can be culled, and/or the environment cleaned (***V*** set to 0).

Although we have developed a full multispecies model, in order to focus on the effects of population structure, we limit our consideration to a single species of farmed livestock, namely ducks [51], [52]. We do examine different parameter values, which cover the case of chickens as well [53]. We also treat the total population size as fixed at first. Population size becomes variable as animals move through the network, increasing as animals move in from other patches, as well as decreasing due to mortality and animals moving out of patches. We did this so as not to confound the results of spatial structure with those of population size. We generally examine equal initial population sizes, again to focus our results on structure; but we have looked a few key cases with mixed population sizes.

## Results

**Figure 2:**
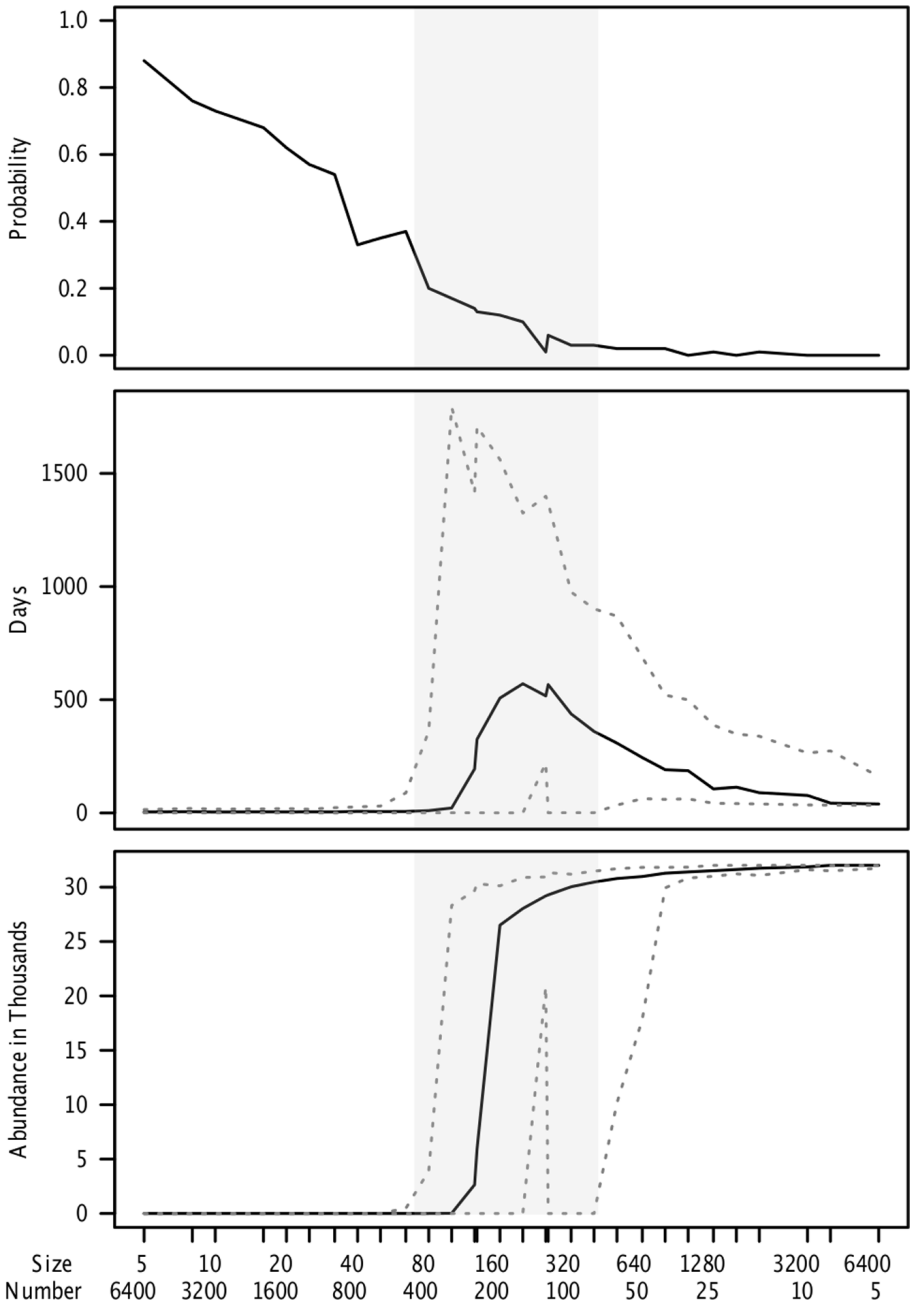
For a fixed total single species total population size 32,000, without non-influenza mortality (μ = 0), the effect of changing local patch size and patch number on (A) frequency of epidemic failure, (B) median length of epidemic in days, and (C) median total number of animals infected over 100 simulations. Dotted lines represent the empirical 97.5% and 2.5% percentiles, creating a 95% bootstrap confidence interval. Other parameters are as in Table 1, including environmental transmission, except α = 0.1111, and ω = 0.03, without any infection control program. Gray area represents parameter region where 1<R_0_<6 within a patch.

Our key finding (Figure 2) is that persistence of A/H5N1 is highly dependent on farm size. For very small patches, the within-patch R_0_ of the pathogen is substantially less than one and most epidemics fail, i.e. there are no cases of secondary transmission. This is true across different population structures (Figures S1–S5). The qualitative result is also robust to different parameter values (Figures S6–S9) [51]–[53]. Although truly random networks can require very large patches for persistence, this may be because the initial two infected hosts frequently land in patches that are completely disconnected from the rest of the network, and it is only once the network is small enough that the infection can actually spread throughout the network (Figure S2). In contrast, the transitions are very stark for a pure nearest neighbor network, the other extreme of the Watts-Strogatz model (Figure S1). The results are qualitatively robust for the topologically very different Barabasi network (Figure S3), but this does not alter the general qualitative conclusions. Including a few larger farms in a matrix of small farms does not alter the results very much until there is a gigantic farm, which reduces the variability in outcomes (Figure S4 & S5).

**Figure 3:**
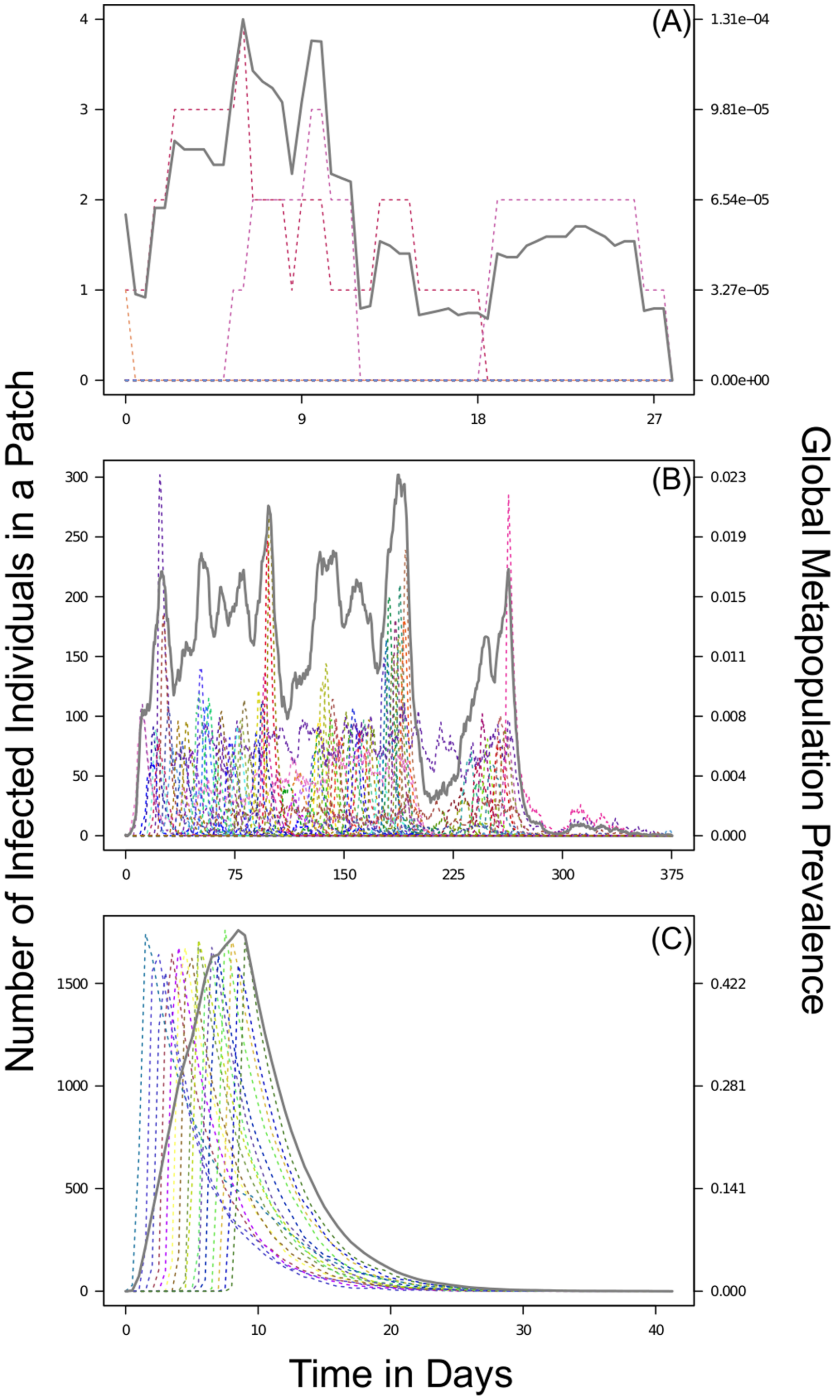
Dynamics over time of for three different simulation runs, solid grey line represents global prevalence of infection across all patches (farms and markets), colored dashed lines represent abundance of infected individuals within each patch (farm and market). (A) 1280 patches of size 25, longest simulation run, note only three patches infected, (B) 128 patches of size 250, random simulation run, note global prevalence always less than 2.5%, (C) 16 patches of size 2000, random simulation run, note near deterministic similarity of epidemic in each patch.

At low patch sizes, if there are some cases of secondary transmission, the epidemic dies out rapidly due to stochasticity (e.g., Figure 3a). For very large patches, the within-patch R_0_ of the pathogen is so high that the epidemic rapidly burns through the population, and as long as the network is sufficiently connected that all patches become infected, they all experience a full epidemic (e.g., Figure 3c). In instances where patches are of moderate size however, resulting in R_0_ values between 1 and approximately 6, patches experience asynchronous local mini-epidemics (e.g., Figure 3b), which prolong the global epidemic. However in these cases, the epidemic doesn’t necessarily infect a large portion of the population, and if pathogenicity is low, may not cause noticeable outbreaks. Variation in the rates of direct transmission, mortality, and recovery, as well as the details of environmental transmission, can alter the pathogen’s ideal patch size, but does not change the fundamental result that a metapopulation of moderate sized farms is best for persistence. Simple mixed initial population size models, with either one (Figure S4) or five (Figure S5) larger populations, and several smaller moderately sized populations (N = 250) do not alter the conclusions until these larger populations are quite large (>5000) relative to the total population size. Even then, the effect is more to reduce the variability, rather than a strong alteration of the mean. This highlights the role that the moderate size farms have in allowing the epidemic to smolder, even if there are a few large epidemics in the system without any control measures.

**Figure 4:**
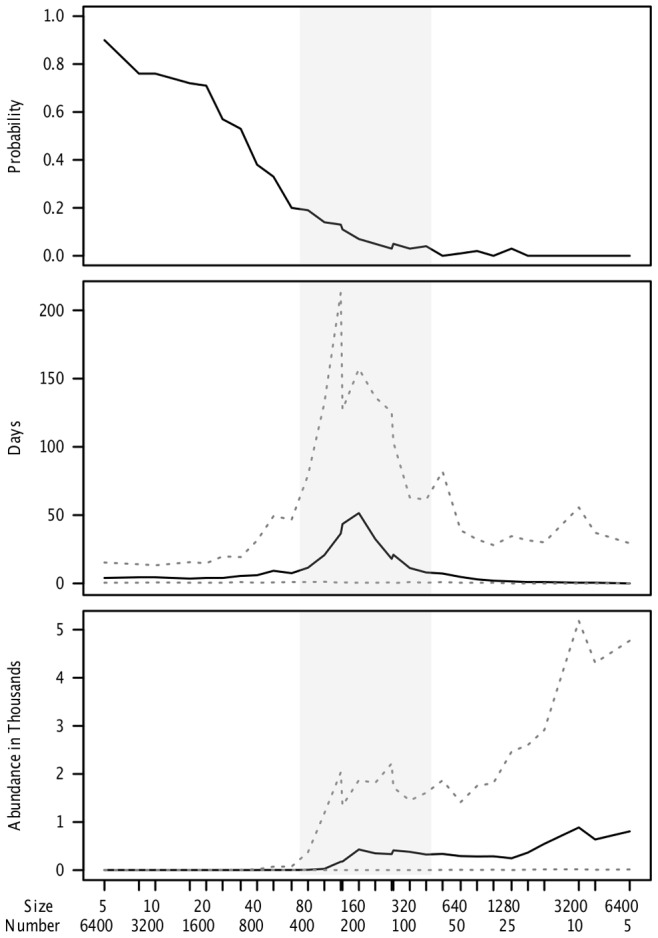
For a fixed total single species total population size (32,000), without non-influenza mortality (μ = 0), the effect of changing local patch size and patch number on (A) frequency of epidemic failure, (B) median length of epidemic in days, and (C) median total number of animals infected over 100 simulations. Dotted lines represent the empirical 97.5% and 2.5% percentiles, creating a 95% bootstrap confidence interval. Here with control measures implemented, (π_Report_ = 0.9, π_Detect_ = 0.9, τ_Crit_ = 1, I_Crit_ = 5). Gray area represents parameter region where 1<R_0_<6 within a patch.

In Figure 4, we implement a control program, namely a passive surveillance program that culls animals and cleans the environment when influenza is detected. While these measures can substantially reduce the total number of individuals infected in larger patches, they are ineffective at curtailing persistence in a network of moderate sized populations. Figure S9 demonstrates that our conclusions are general, although the quantitative details depend on exact parameters.

## Discussion

Our model demonstrates a strong role of farm size in the risk of highly pathogenic avian influenza (HPAI) outbreaks. Our results suggest that true small-scale subsistence farming at low densities has a low risk of HPAI outbreaks, and since the risk of outbreaks is reduced, the risk of spillover to people should be reduced. These results run counter to a number of previous studies which have linked human cases of highly pathogenic A/H5N1 influenza to small-scale, backyard poultry operations and proposed that this is a major risk factor for future spillover events [54]–[57], however, the small and moderate scales (discussed below) in our model are rarely separated in the empirical literature, as both fall into the FAO/OIE sector 4 designation [58], [59]. In most cases, these studies consist of investigations of human cases, outbreaks, or policy recommendations based on epidemiological analyses of these. Thus results here suggest that patchily distributed very small-scale low density poultry production are insufficient to sustain epidemics, and a fragmented trade network may instead reduce the probability of sustained transmission.

As human density increases, patches of poultry likely become larger – either from the development of modest poultry production by local entrepreneurs, or because individual family flocks intermingle so extensively to become a single flock in high-density environments, or from a mix of these scenarios. In this case, a smoldering epidemic might allow an HPAI to persist without ever causing enough livestock mortality to enable effective intervention. The countries that have had the most reported H5N1 cases share these characteristics in common, despite geographic distance. Indonesia, Vietnam, and Egypt have high human population density, where people raise poultry both for subsistence and for income, but on a moderately small, household scale [58].

Our modeling work suggests that truly large-scale poultry production, such as Concentrated Animal Feeding Operations (CAFOs), could reduce the persistence of A/H5N1 by reducing the likelihood of undetected and under-reported smoldering epidemics and thus reduce the risk of spillover to humans. A critical part of this would be a control program that succeeds at identifying outbreaks in these farms sufficiently early to provide a public health benefit; otherwise smoldering persistence would become raging epidemics. Additionally, it would need to be clear that the advantages from making epidemics more detectable offset the disadvantage of any increase in the size of epidemics for the risk of spillover to humans. A small number of studies have highlighted the lack of biosecurity in intensive poultry farms [60], [61]. However, these studies are focused on developed countries, where there is an expectation of reliable, advanced biosecurity, and when lapses are identified, they are considered significant. In developing countries, the difference in biosecurity between industrial farms and backyard production is likely far larger, even if neither is as biosecure as in developed countries. Here, there are potential practical reasons for a protective role of large-scale farms. In these developing country large-scale farms, there is likely to be less trading in and out of the population and far more surveillance for mortality than in small backyard flocks. Therefore an HPAI outbreak would be large and noticeable and therefore more likely to be subject to control measures such as the de-flocking or mass vaccination strategies used widely in China, southeast Asia and Egypt [58].

Comparing the results from different network structures, it is clear that this is an important factor, particularly for systems of larger farms. A nearest neighbor network (no long-distance links across the network) produced results almost similar to culling. However, increasing farm size did reduce persistence in all cases, albeit with different patterns. Yet the critical threshold farm-size for enabling persistence was similar across network patterns, demonstrating that the within patch R_0_ is the most important factor for this threshold. Unfortunately, the detailed comparative data on real trade networks, and poultry farming practices across countries that would be needed to test this model are currently lacking, or relatively inaccessible proprietary data, although we are seeking to collaborate with other groups and nations to obtain this data, as well as working on our own empirical research.

It is important to note that our model can explain the persistence of A/H5N1 influenza without invoking repeated introductions from wildlife, or domestic, reservoirs. Wild waterfowl harbor a diversity of influenza strains, including A/H5N1, which has been reported from wild birds in Egypt [62], South Asia [32], China [2], [34], [63], Europe [64] and Africa [65], [66]. However, while there is evidence for a role of wild birds in the spread and introduction of A/H5N1 [31], it is not clear what role infected wild birds play in persistence of HPAIs generally. Our modeling suggests that the wild birds are not required for persistence, in line with a recent empirical review by Gauthier-Clerc [67].

Culling remains the most widespread and commonly used approach for dealing with HPAI infections in endemic countries [58]. Our model demonstrates the effectiveness of culling in reducing the number of infected individuals in large poultry populations. However, neither culling, nor culling and cleaning of the environment was able to reduce persistence of influenza in our simulations for metapopulations of moderate sized farms. Our model results suggest that changes to the type of farms present within endemic countries would have a more significant impact on the persistence of HPAI, and therefore the long-term effectiveness of control programs. In essence, once the protein demands of country require the intensification of poultry production beyond subsistence, from an emerging disease risk prospective it may be best to move to CAFOs as quickly as possible. A preliminary suggestion in terms of network structure would be to subdivide the farm to market chain into as many small separate networks as possible, but this may not be feasible.

In this study, we did not examine the efficacy of vaccination as a control strategy because of the complexity of escape mutations, and problems with its long-term use as a control measure [20], [68], [69]. Therefore, a model that proposed to examine a vaccination control strategy would have to include strain variation and antigenic selection (e.g. [70]), which is beyond the scope of this work. Future work should investigate the ability of this model to fit observed dynamics in empirical systems, the potential effects of variability of initial farm size, and alternate network structure [71], both empirical and theoretical. This model could be helpful in optimizing control efforts could potentially yield tremendous benefits to the poultry industry, as the virus depresses exports in affected countries, with China and Thailand alone losing $900 million due A/H5N1 from to 2003 to 2005 [72].

Our study demonstrates that moderate size patches of poultry may substantially contribute to the persistence of influenza in countries where such production is a dominant force in the poultry production system. Although passive surveillance and culling may reduce prevalence, and infection burden, and thus risk of spillover it is unlikely to eliminate influenza from these countries. Development of more bio-secure, intensively monitored, larger scale commercial poultry production may be the best route to risk reduction in countries with substantial need for intensive poultry production.

## Supporting Information

Figure S1 **Alternate Watts-Strogatz network, here re-wiring probability ρ = 0, thus only the two nearest neighbors in either direction are connected, and there are no random long distance connections across the network.** For a fixed total single species total population size 32,000, without non-influenza mortality (μ = 0), the effect of changing local patch size and patch number on (A) frequency of epidemic failure, (B) median length of epidemic in days, and (C) median total number of animals infected over 100 simulations. Dotted lines represent the empirical 97.5% and 2.5% percentiles, creating a 95% bootstrap confidence interval. Other parameters are as in Table 1, including environmental transmission, except α = 0.1111, and ω = 0.03, without any infection control program. Gray area represents parameter region where 1< R_0_<6 within a patch.
(TIF)

Figure S2 **Alternate Watts-Strogatz network, here re-wiring probability ρ = 0, which transforms the network to an essentially randomly wired network.** This can create sub-networks that are disconnected from the majority of the network leading to high variability in simulation results, and pushing the persistence area to very large farm sizes. For a fixed total single species total population size 32,000, without non-influenza mortality (μ = 0), the effect of changing local patch size and patch number on (A) frequency of epidemic failure, (B) median length of epidemic in days, and (C) median total number of animals infected over 100 simulations. Dotted lines represent the empirical 97.5% and 2.5% percentiles, creating a 95% bootstrap confidence interval. Other parameters are as in Table 1, including environmental transmission, except α = 0.1111, and ω = 0.03, without any infection control program. Gray area represents parameter region where 1< R_0_<6 within a patch.
(TIF)

Figure S3 **Alternate random network, here a non-directional Barabasi network** [**48**] **, with a zero appeal of 1 and a power of preferential attachment of 0.5.** For a fixed total single species total population size 32,000, without non-influenza mortality (μ = 0), the effect of changing local patch size and patch number on (A) frequency of epidemic failure, (B) median length of epidemic in days, and (C) median total number of animals infected over 100 simulations. Dotted lines represent the empirical 97.5% and 2.5% percentiles, creating a 95% bootstrap confidence interval. Other parameters are as in Table 1, including environmental transmission, except α = 0.1111, and ω = 0.03, without any infection control program. Gray area represents parameter region where 1< R_0_<6 within a patch.
(TIF)

Figure S4 **Standard small world network (ρ = 0.6), but there is now one larger farm of variable size.** The top row of the x-axis is the size of the larger patch, while the bottom row is the number of smaller patches, all of size 250 hosts. Thus the far left of the graph is the same as for Figure 1 in the main text. For a fixed total single species total population size 32,000, without non-influenza mortality (μ = 0), the effect of changing local patch size and patch number on (A) frequency of epidemic failure, (B) median length of epidemic in days, and (C) median total number of animals infected over 100 simulations. Dotted lines represent the empirical 97.5% and 2.5% percentiles, creating a 95% bootstrap confidence interval. Other parameters are as in Table 1, including environmental transmission, except α = 0.1111, and ω = 0.03, without any infection control program.
(TIF)

Figure S5 **Standard small world network (ρ = 0.6), but there is now five larger farms of variable size.** The top row of the x-axis is the size of each of the five larger patches, while the bottom row is the number of smaller patches, all of size 250 hosts. Thus the far left of the graph is the same as for Figure 1 in the main text. For a fixed total single species total population size 32,000, without non-influenza mortality (μ = 0), the effect of changing local patch size and patch number on (A) frequency of epidemic failure, (B) median length of epidemic in days, and (C) median total number of animals infected over 100 simulations. Dotted lines represent the empirical 97.5% and 2.5% percentiles, creating a 95% bootstrap confidence interval. Other parameters are as in Table 1, including environmental transmission, except α = 0.1111, and ω = 0.03, without any infection control program.
(TIF)

Figure S6 **Alternate transmission parameters that more closely resemble H5N1 infections in chickens with no recovery and faster mortality** [**51**] **.** For a fixed total single species total population size 32,000, without non-influenza mortality (μ = 0), the effect of changing local patch size and patch number on (A) frequency of epidemic failure, (B) median length of epidemic in days, and (C) median total number of animals infected over 100 simulations. Dotted lines represent the empirical 97.5% and 2.5% percentiles, creating a 95% bootstrap confidence interval. Other parameters are as in Table 1, including environmental transmission, except β = 0.0081, γ = 0, α = 0.32, and ω = 0.05, without any infection control program.
(TIF)

Figure S7 **Alternate transmission parameters that emphasize environmental transmission more and direct transmission less, with higher mortality.** For a fixed total single species total population size 32,000, without non-influenza mortality (μ = 0), the effect of changing local patch size and patch number on (A) frequency of epidemic failure, (B) median length of epidemic in days, and (C) median total number of animals infected over 100 simulations. Dotted lines represent the empirical 97.5% and 2.5% percentiles, creating a 95% bootstrap confidence interval. Other parameters are as in Table 1, including environmental transmission, except β = 0.003, υ = 0.002, α = 0.222, and ω = 0.143, without any infection control program.
(TIF)

Figure S8 **Alternate transmission parameters without environmental transmission.** For a fixed total single species total population size 32,000, without non-influenza mortality (μ = 0), the effect of changing local patch size and patch number on (A) frequency of epidemic failure, (B) median length of epidemic in days, and (C) median total number of animals infected over 100 simulations. Dotted lines represent the empirical 97.5% and 2.5% percentiles, creating a 95% bootstrap confidence interval. Other parameters are as in Table 1, excluding environmental transmission, i.e. υ = 0.0, without any infection control program.
(TIF)

Figure S9 **Less effective control program, π_Report_ = 0.1, not 0.9.** For a fixed total single species total population size (32,000), without non-influenza mortality (μ = 0), the effect of changing local patch size and patch number on (A) frequency of epidemic failure, (B) median length of epidemic in days, and (C) median total number of animals infected over 100 simulations. Dotted lines represent the empirical 97.5% and 2.5% percentiles, creating a 95% bootstrap confidence interval. Here with control measures implemented, (π_Report_ = 0.1, π_Detect_ = 0.9, τ_Crit_ = 1, I_Crit_ = 5).
(TIF)

## References

[1] Subbarao K, Klimov A, Katz J, Regnery H, Lim W, et al (1998) Characterization of an avian influenza A (H5N1) virus isolated from a child with a fatal respiratory illness. Science 279: 393–396. 9430591 10.1126/science.279.5349.393

[2] Chen H, Smith GJD, Zhang SY, Qin K, Wang J, et al (2005) H5N1 virus outbreak in migratory waterfowl. Nature 436: 191–192. 16007072 10.1038/nature03974

[3] Breed AC, Harris K, Hesterberg U, Gould G, Londt BZ, et al (2010) Surveillance for Avian Influenza in Wild Birds in the European Union in 2007. Avian Diseases 54: 399–404. 20521669 10.1637/8950-053109-Reg.1

[4] Lebarbenchon C, Albespy F, Brochet AL, Grandhomme V, Renaud F, et al.. (2009) Spread of Avian Influenza Viruses by Common Teal (Anas crecca) in Europe. PLoS ONE 4. 10.1371/journal.pone.0007289 PMC2750755 19802387

[5] Zohari S, Gyarmati P, Thoren P, Czifra G, Brorjer C, et al (2008) Genetic characterization of the NS gene indicates co-circulation of two sub-lineages of highly pathogenic avian influenza virus of H5N1 subtype in Northern Europe in 2006. Virus Genes 36: 117–125. 18172752 10.1007/s11262-007-0188-7

[6] Nagy A, Vostinakova V, Pindova Z, Hornickova J, Cernikova L, et al (2009) Molecular and phylogenetic analysis of the H5N1 avian influenza virus caused the first highly pathogenic avian influenza outbreak in poultry in the Czech Republic in 2007. Veterinary Microbiology 133: 257–263. 18789611 10.1016/j.vetmic.2008.07.013

[7] Ray K, Potdar VA, Cherian SS, Pawar SD, Jadhav SM, et al (2008) Characterization of the complete genome of influenza A (H5N1) virus isolated during the 2006 outbreak in poultry in India. Virus Genes 36: 345–353. 18214665 10.1007/s11262-007-0195-8

[8] Aly MM, Arafa A, Hassan MK (2008) Epidemiological Findings of Outbreaks of Disease Caused by Highly Pathogenic H5N1 Avian Influenza Virus in Poultry in Egypt During 2006. Avian Diseases 52: 269–277. 18646456 10.1637/8166-103007-Reg.1

[9] Truscott J, Garske T, Chis-Ster I, Guitian J, Pfeiffer D, et al (2007) Control of a highly pathogenic H5N1 avian influenza outbreak in the GB poultry flock. Proceedings of the Royal Society B-Biological Sciences 274: 2287–2295. 10.1098/rspb.2007.0542 PMC2288522 17644506

[10] Viseshakul N, Thanawongnuwech R, Amonsin A, Suradhat S, Payungporn S, et al (2004) The genome sequence analysis of H5N1 avian influenza A virus isolated from the outbreak among poultry populations in Thailand. Virology 328: 169–176. 15464837 10.1016/j.virol.2004.06.045

[11] Shortridge KF (1999) Poultry and the influenza H5N1 outbreak in Hong Kong, 1997. Abridged chronology and virus isolation. Vaccine 17: S26–S29. 10471177 10.1016/s0264-410x(99)00102-4

[12] Monne I, Joannis TM, Fusaro A, De Benedictis P, Cattoli G, et al (2008) Reassortant avian influenza virus (H5N1) in poultry, Nigeria, 2007 (vol 14, pg 637, 2008). Emerging Infectious Diseases 14: 865–865. 10.3201/eid1404.071178 PMC2570913 18394282

[13] Fasina FO, Rivas AL, Bisschop SPR, Stegeman AJ, Hernandez JA (2011) Identification of risk factors associated with highly pathogenic avian influenza H5N1 virus infection in poultry farms, in Nigeria during the epidemic of 2006–2007. Preventive Veterinary Medicine 98: 204–208. 21146235 10.1016/j.prevetmed.2010.11.007

[14] Kandeel A, Manoncourt S, Abd el Kareem E, Ahmed ANM, El-Refaie S, et al (2010) Zoonotic Transmission of Avian Influenza Virus (H5N1), Egypt, 2006–2009. Emerging Infectious Diseases 16: 1101–1107. 20587181 10.3201/eid1607.091695 PMC3321902

[15] Hien ND, Ha NH, Van NT, Ha NTM, Lien TTM, et al (2009) Human Infection with Highly Pathogenic Avian Influenza Virus (H5N1) in Northern Vietnam, 2004–2005. Emerging Infectious Diseases 15: 19–23. 19116044 10.3201/eid1501.080073 PMC2660684

[16] Barral M, Alvarez V, Juste RA, Agirre I, Inchausti I (2008) First case of highly pathogenic H5N1 avian influenza virus in Spain. Bmc Veterinary Research 4. 10.1186/1746-6148-4-50 PMC2621144 19077185

[17] WHO (2012) Epidemic and Pandemic Alert and Response: Avian Influenza. WHO.

[18] Berns KI, Casadevall A, Cohen ML, Ehrlich SA, Enquist LW, et al (2012) Adaptations of Avian Flu Virus Are a Cause for Concern. Science 335: 660–661. 22294736 10.1126/science.1217994

[19] EMPRES (2009) Global Animal Disease Information System. FAO.

[20] Webster RG, Bean WJ, Gorman OT, Chambers TM, Kawaoka Y (1992) Evolution and ecology of influenza A viruses. Microbiological Reviews 56: 152–179. 1579108 10.1128/mr.56.1.152-179.1992 PMC372859

[21] Gilbert M, Xiao XM, Pfeiffer DU, Epprecht M, Boles S, et al (2008) Mapping H5N1 highly pathogenic avian influenza risk in Southeast Asia. Proceedings of the National Academy of Sciences of the United States of America 105: 4769–4774. 18362346 10.1073/pnas.0710581105 PMC2290786

[22] Guan Y, Smith GJD, Webby R, Webster RG (2009) Molecular epidemiology of H5N1 avian influenza. Revue Scientifique Et Technique-Office International Des Epizooties 28: 39–47. 10.20506/rst.28.1.1868 19618617

[23] OIE (2012) Update on Avian Influenza in animals (Type H5).

[24] Bavinck V, Bouma A, van Boven M, Bos MEH, Stassen E, et al (2009) The role of backyard poultry flocks in the epidemic of highly pathogenic avian influenza virus (H7N7) in the Netherlands in 2003. Preventive Veterinary Medicine 88: 247–254. 19178969 10.1016/j.prevetmed.2008.10.007

[25] Biswas PK, Christensen JP, Ahmed SSU, Barua H, Das A, et al (2008) Avian Influenza Outbreaks in Chickens, Bangladesh. Emerging Infectious Diseases 14: 1909–1912. 19046518 10.3201/eid1412.071567 PMC2634614

[26] Hafez MH, Arafa A, Abdelwhab EM, Selim A, Khoulosy SG, et al (2010) Avian influenza H5N1 virus infections in vaccinated commercial and backyard poultry in Egypt. Poultry Science 89: 1609–1613. 10.3382/ps.2010-00708 20634514

[27] Van Kerkhove MD, Ly S, Holl D, Guitian J, Mangtani P, et al (2008) Frequency and patterns of contact with domestic poultry and potential risk of H5N1 transmission to humans living in rural Cambodia. Influenza and Other Respiratory Viruses 2: 155–163. 19453420 10.1111/j.1750-2659.2008.00052.x PMC4941898

[28] Martin V, Pfeiffer DU, Zhou XY, Xiao XM, Prosser DJ, et al.. (2011) Spatial distribution and risk factors of Highly Pathogenic Avian Influenza (HPAI) H5N1 in China. PLoS Pathog 7. 10.1371/journal.ppat.1001308 PMC3048366 21408202

[29] Gilbert M, Xiao XM, Chaitaweesub P, Kalpravidh W, Premashthira S, et al (2007) Avian influenza, domestic ducks and rice agriculture in Thailand. Agriculture Ecosystems & Environment 119: 409–415. 10.1016/j.agee.2006.09.001 PMC2311503 18418464

[30] Reperant LA, Fuckar NS, Osterhaus ADME, Dobson AP, Kuiken T (2010) Spatial and temporal association of outbreaks of H5N1 influenza virus infection in wild birds with the 0 degrees C isotherm. PLoS Pathog 6: e1000854. 20386716 10.1371/journal.ppat.1000854 PMC2851735

[31] Kilpatrick AM, Chmura AA, Gibbons DW, Fleischer RC, Marra PP, et al (2006) Predicting the global spread of H5N1 avian influenza. Proceedings of the National Academy of Sciences of the United States of America 103: 19368–19373. 17158217 10.1073/pnas.0609227103 PMC1748232

[32] Gilbert M, Newman SH, Takekawa JY, Loth L, Biradar C, et al (2010) Flying Over an Infected Landscape: Distribution of Highly Pathogenic Avian Influenza H5N1 Risk in South Asia and Satellite Tracking of Wild Waterfowl. Ecohealth 7: 448–458. 21267626 10.1007/s10393-010-0672-8 PMC3166606

[33] Gilbert M, Xiao XM, Domenech J, Lubroth J, Martin V, et al (2006) Anatidae migration in the western palearctic and spread of highly pathogenic avian influenza H5N1 virus. Emerging Infectious Diseases 12: 1650–1656. 17283613 10.3201/eid1211.060223 PMC3372333

[34] Takekawa JY, Newman SH, Xiao XM, Prosser DJ, Spragens KA, et al (2010) Migration of Waterfowl in the East Asian Flyway and Spatial Relationship to HPAI H5N1 Outbreaks. Avian Diseases 54: 466–476. 20521681 10.1637/8914-043009-Reg.1 PMC4878034

[35] Williams RAJ, Xiao XM, Peterson AT (2011) Continent-wide association of H5N1 outbreaks in wild and domestic birds in Europe. Geospatial Health 5: 247–253. 21590675 10.4081/gh.2011.177 PMC4868044

[36] Xiao XM, Gilbert M, Slingenbergh J, Lei F, Boles S (2007) Remote sensing, ecological variables, and wild bird migration related to outbreaks of highly pathogenic H5N1 avian influenza. Journal of Wildlife Diseases 43: S40–S46. 28813587 PMC2735754

[37] Hanski I, Gilpin ME (1997) Metapopulation biology: ecology, genetics, and evolution. San Diego: Academic Press.

[38] Durrett R, Levin S (1994) The Importance Of Being Discrete (And Spatial). Theoretical Population Biology 46: 363–394.

[39] Moore SM, Manore CA, Bokil VA, Borer ET, Hosseini PR (2011) Spatiotemporal Model of Barley and Cereal Yellow Dwarf Virus Transmission Dynamics with Seasonality and Plant Competition. Bulletin of Mathematical Biology 73: 2707–2730. 21505932 10.1007/s11538-011-9654-4

[40] Thrall PH, Burdon JJ (1999) The spatial scale of pathogen dispersal: Consequences for disease dynamics and persistence. Evolutionary Ecology Research 1: 681–701.

[41] Ellner SP, McCauley E, Kendall BE, Briggs CJ, Hosseini PR, et al (2001) Habitat structure and population persistence in an experimental community. Nature 412: 538–543. 11484053 10.1038/35087580

[42] Ferrari MJ, Djibo A, Grais RF, Bharti N, Grenfell BT, et al (2010) Rural-urban gradient in seasonal forcing of measles transmission in Niger. Proceedings of the Royal Society B-Biological Sciences 277: 2775–2782. 10.1098/rspb.2010.0536 PMC2981991 20427338

[43] Plowright RK, Foley P, Field HE, Dobson AP, Foley JE, et al (2011) Urban habituation, ecological connectivity and epidemic dampening: The emergence of Hendra virus from flying foxes (*Pteropus* species). Proceedings of the Royal Society B-Biological Sciences 278: 3703–3712. 10.1098/rspb.2011.0522 PMC3203503 21561971

[44] Beyer HL, Hampson K, Lembo T, Cleaveland S, Kaare M, et al (2011) Metapopulation dynamics of rabies and the efficacy of vaccination. Proceedings of the Royal Society B-Biological Sciences 278: 2182–2190. 10.1098/rspb.2010.2312 PMC3107631 21159675

[45] Courcoul A, Ezanno P (2010) Modelling the spread of Bovine Viral Diarrhoea Virus (BVDV) in a managed metapopulation of cattle herds. Veterinary Microbiology 142: 119–128. 19875250 10.1016/j.vetmic.2009.09.052

[46] Doob JL (1942) Topics in the theory of Markoff chains. Transactions of the American Mathematical Society 52: 37–64.

[47] Gillespie DT (1977) Exact stochastic simulation of coupled chemical reactions. The journal of physical chemistry 81: 2340–2361.

[48] Breban R, Drake JM, Stallknecht DE, Rohani P (2009) The Role of Environmental Transmission in Recurrent Avian Influenza Epidemics. Plos Computational Biology 5. 10.1371/journal.pcbi.1000346 PMC2660440 19360126

[49] Watts DJ, Strogatz SH (1998) Collective dynamics of ‘small-world’ networks. Nature 393: 440–442. 9623998 10.1038/30918

[50] Barabasi AL, Albert R (1999) Emergence of scaling in random networks. Science 286: 509–512. 10521342 10.1126/science.286.5439.509

[51] Sakoda Y, Sugar S, Batchluun D, Erdene-Ochir TO, Okamatsu M, et al (2010) Characterization of H5N1 highly pathogenic avian influenza virus strains isolated from migratory waterfowl in Mongolia on the way back from the southern Asia to their northern territory. Virology 406: 88–94. 20673942 10.1016/j.virol.2010.07.007

[52] Phuong DQ, Dung NT, Jorgensen PH, Handberg KJ, Vinh NT, et al (2011) Susceptibility of Muscovy (Cairina Moschata) and mallard ducks (Anas Platyrhynchos) to experimental infections by different genotypes of H5N1 avian influenza viruses. Veterinary Microbiology 148: 168–174. 20943331 10.1016/j.vetmic.2010.09.007

[53] Bouma A, Claassen I, Natih K, Klinkenberg D, Donnelly CA, et al.. (2009) Estimation of Transmission Parameters of H5N1 Avian Influenza Virus in Chickens. Plos Pathogens 5. 10.1371/journal.ppat.1000281 PMC2627927 19180190

[54] Biswas PK, Christensen JP, Ahmed SSU, Das A, Rahman MH, et al (2009) Risk for Infection with Highly Pathogenic Avian Influenza Virus (H5N1) in Backyard Chickens, Bangladesh. Emerging Infectious Diseases 15: 1931–1936. 19961672 10.3201/eid1512.090643 PMC3044532

[55] Cristalli A, Capua I (2007) Practical problems in controlling H5N1 high pathogenicity avian influenza at village level in Vietnam and introduction of biosecurity measures. Avian Diseases 51: 461–462. 17494607 10.1637/7564-033106R.1

[56] Fielding R, Bich TH, Quang LN, Lam WWT, Leung GM, et al (2007) Live poultry exposures, Hong Kong and Hanoi, 2006. Emerging Infectious Diseases 13: 1065–1067. 18214181 10.3201/eid1307.061031 PMC2878218

[57] Tiensin T, Nielen M, Vernooij H, Songserm T, Kalpravidh W, et al (2007) Transmission of the highly pathogenic avian influenza virus H5N1 within flocks during the 2004 epidemic in Thailand. Journal of Infectious Diseases 196: 1679–1684. 18008253 10.1086/522007

[58] FAO (2005) A Global Strategy for the Progressive Control of Highly Pathogenic Avian Influenza (HPAI). Food and Agriculture Organization, World Health Organization.

[59] Hosny FA (2006) Egypt: Poultry sector country review. FAO. 47 p.

[60] Graham JP, Leibler JH, Price LB, Otte JM, Pfeiffer DU, et al (2008) The animal-human interface and infectious disease in industrial food animal production: Rethinking biosecurity and biocontainment. Public Health Reports 123: 282–299. 19006971 10.1177/003335490812300309 PMC2289982

[61] Leibler JH, Otte J, Roland-Holst D, Pfeiffer DU, Magalhaes RS, et al (2009) Industrial Food Animal Production and Global Health Risks: Exploring the Ecosystems and Economics of Avian Influenza. Ecohealth 6: 58–70. 19437076 10.1007/s10393-009-0226-0 PMC7087879

[62] Saad MD, Ahmed LS, Gamal-Eldein MA, Fouda MK, Khalil FM, et al (2007) Possible avian influenza (H5N1) from migratory bird, Egypt. Emerging Infectious Diseases 13: 1120–1121. 18214200 10.3201/eid1307.061222 PMC2878221

[63] Liu J, Xiao H, Lei F, Zhu Q, Qin K, et al (2005) Highly pathogenic H5N1 influenza virus infection in migratory birds. Science 309: 1206–1206. 16000410 10.1126/science.1115273

[64] Szeleczky Z, Dan A, Ursu K, Ivanics E, Kiss I, et al (2009) Four different sublineages of highly pathogenic avian influenza H5N1 introduced in Hungary in 2006–2007. Veterinary Microbiology 139: 24–33. 19520524 10.1016/j.vetmic.2009.04.017

[65] Alexander DJ (2007) Summary of avian influenza activity in Europe, Asia, Africa, and Australasia, 2002–2006. Avian Diseases 51: 161–166. 17494548 10.1637/7602-041306R.1

[66] Cumming GS, Caron A, Abolnik C, Cattoli G, Bruinzeel LW, et al (2012) The Ecology of Influenza A Viruses in Wild Birds in Southern Africa. Ecohealth 8: 4–13. 10.1007/s10393-011-0684-z 21516374

[67] Gauthier-Clerc M, Lebarbenchon C, Thomas F (2007) Recent expansion of highly pathogenic avian influenza H5N1: a critical review. Ibis 149: 202–214.

[68] Lee CW, Senne DA, Suarez DL (2004) Effect of vaccine use in the evolution of Mexican lineage H5N2 avian influenza virus. Journal of Virology 78: 8372–8381. 15254209 10.1128/JVI.78.15.8372-8381.2004 PMC446090

[69] Smith DJ, Lapedes AS, de Jong JC, Bestebroer TM, Rimmelzwaan GF, et al (2004) Mapping the antigenic and genetic evolution of influenza virus. Science 305: 371–376. 15218094 10.1126/science.1097211

[70] Koelle K, Cobey S, Grenfell B, Pascual M (2006) Epochal evolution shapes the phylodynamics of interpandemic influenza A (H3N2) in humans. Science 314: 1898–1903. 17185596 10.1126/science.1132745

[71] Salathé M, Jones JH (2010) Dynamics and Control of Diseases in Networks with Community Structure. PLoS Comput Biol 6: e1000736. 20386735 10.1371/journal.pcbi.1000736 PMC2851561

[72] Nicita A (2008) Avian influenza and the poultry trade. The World Bank.

[73] Sturm-Ramirez KM, Ellis T, Bousfield B, Bissett L, Dyrting K, et al (2004) Reemerging H5N1 influenza viruses in Hong Kong in 2002 are highly pathogenic to ducks. Journal of Virology 78: 4892–4901. 15078970 10.1128/JVI.78.9.4892-4901.2004 PMC387679

[74] Kalthoff D, Breithaupt A, Teifke JP, Globig A, Harder T, et al (2008) Highly pathogenic avian influenza virus (H5N1) in experimentally infected adult mute swans. Emerg Infect Dis 14: 1267–1270. 18680652 10.3201/eid1408.080078 PMC2600380

