# Correction: Metapopulation Dynamics Enable Persistence of Influenza A, Including A/H5N1, in Poultry

**DOI:** 10.1371/annotation/4de9438b-646c-4efd-bf12-62a9baa301e2

**Published:** 2014-01-02

**Authors:** Parviez Rana Hosseini, Trevon Fuller, Ryan Harrigan, Delong Zhao, Carmen Sofia Arriola, Armandoe Gonzalez, Matthew Joshua Miller, Xiangming Xiao, Thomas B. Smith, Jamie Holland Jones, Peter Daszak

The ninth author is listed as Tom B. Smith. His name should instead read Thomas B. Smith.

The following information was missing from the Funding section: "This study was also made possible by the generous support of the American people through the United States Agency for International Development (USAID) Emerging Pandemic Threats PREDICT."

