# Supplementary figures and images for: Metapopulation Dynamics Enable Persistence of Influenza A, Including A/H5N1, in Poultry
Source: PLoS One. 2013 Dec 2;8(12):e80091. doi: 10.1371/journal.pone.0080091 (PMC3846554; doi:10.1371/journal.pone.0080091)

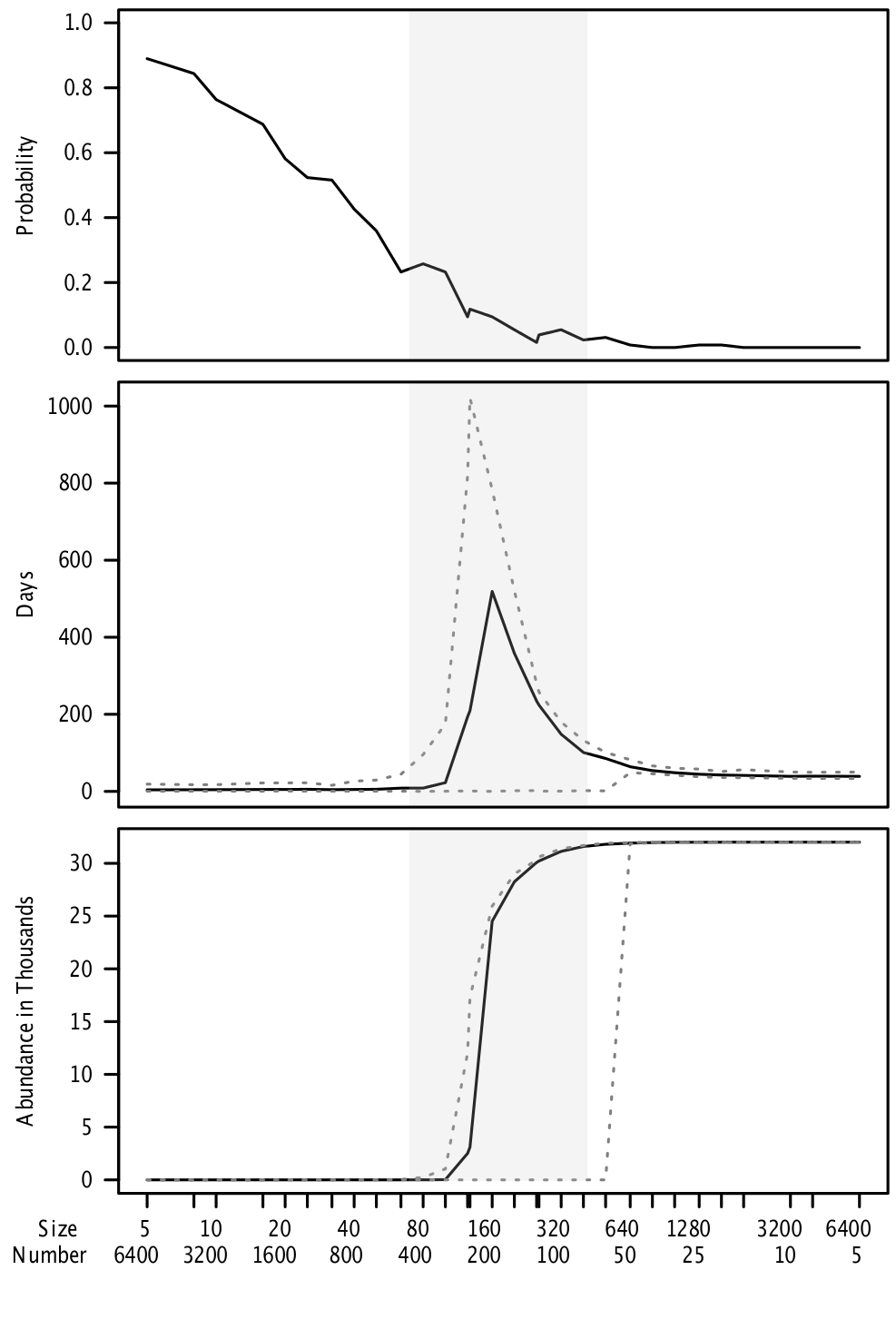

Supplement: Figure S1 — Alternate Watts-Strogatz network, here re-wiring probability ρ = 0, thus only the two nearest neighbors in either direction are connected, and there are no random long distance connections across the network. For a fixed total single species total population size 32,000, without non-influenza mortality (μ = 0), the effect of changing local patch size and patch number on (A) frequency of epidemic failure, (B) median length of epidemic in days, and (C) median total number of animals infected over 100 simulations. Dotted lines represent the empirical 97.5% and 2.5% percentiles, creating a 95% bootstrap confidence interval. Other parameters are as in Table 1, including environmental transmission, except α = 0.1111, and ω = 0.03, without any infection control program. Gray area represents parameter region where 1< R0<6 within a patch. (TIF) [file pone.0080091.s001.tif]

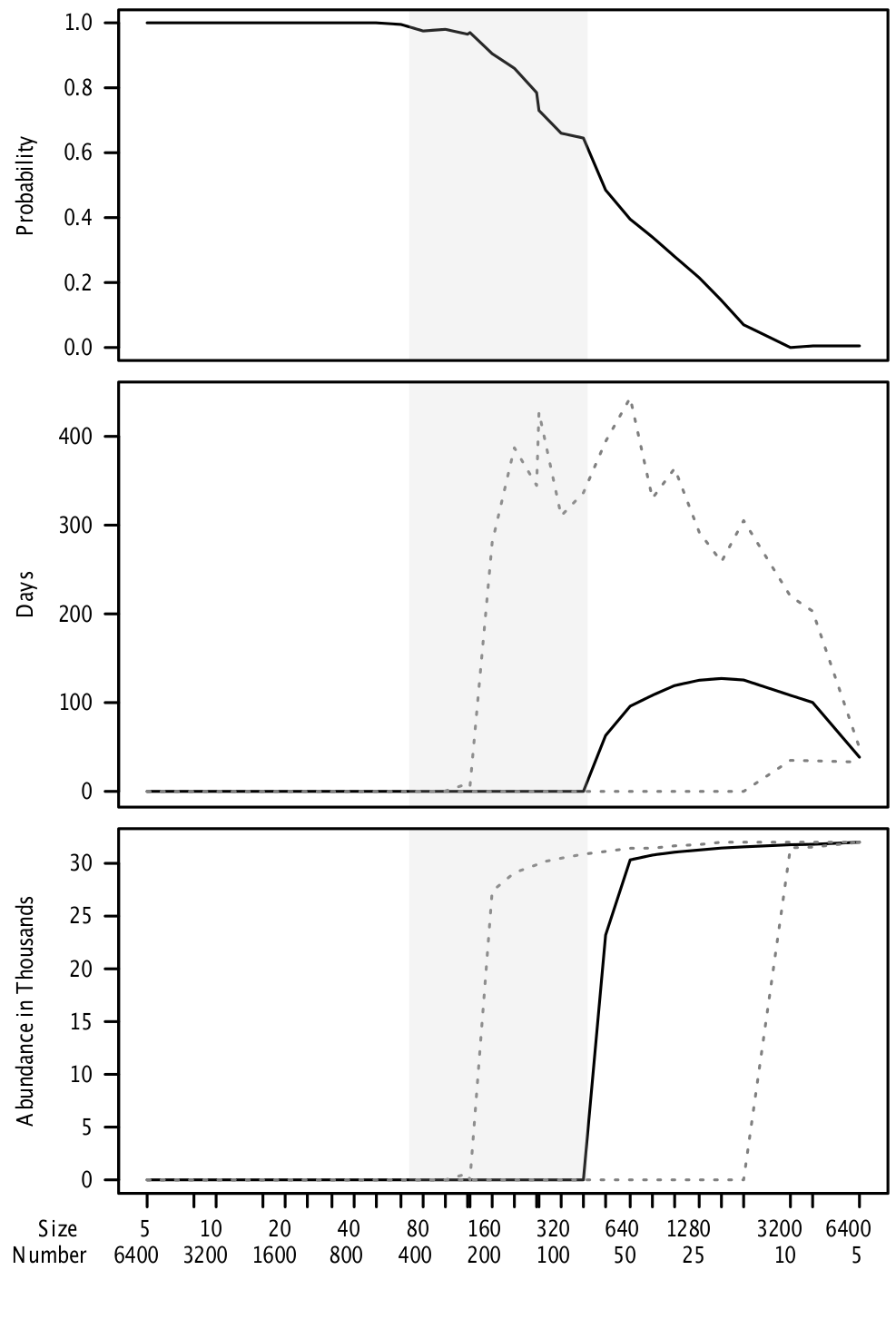

Supplement: Figure S2 — Alternate Watts-Strogatz network, here re-wiring probability ρ = 0, which transforms the network to an essentially randomly wired network. This can create sub-networks that are disconnected from the majority of the network leading to high variability in simulation results, and pushing the persistence area to very large farm sizes. For a fixed total single species total population size 32,000, without non-influenza mortality (μ = 0), the effect of changing local patch size and patch number on (A) frequency of epidemic failure, (B) median length of epidemic in days, and (C) median total number of animals infected over 100 simulations. Dotted lines represent the empirical 97.5% and 2.5% percentiles, creating a 95% bootstrap confidence interval. Other parameters are as in Table 1, including environmental transmission, except α = 0.1111, and ω = 0.03, without any infection control program. Gray area represents parameter region where 1< R0<6 within a patch. (TIF) [file pone.0080091.s002.tif]

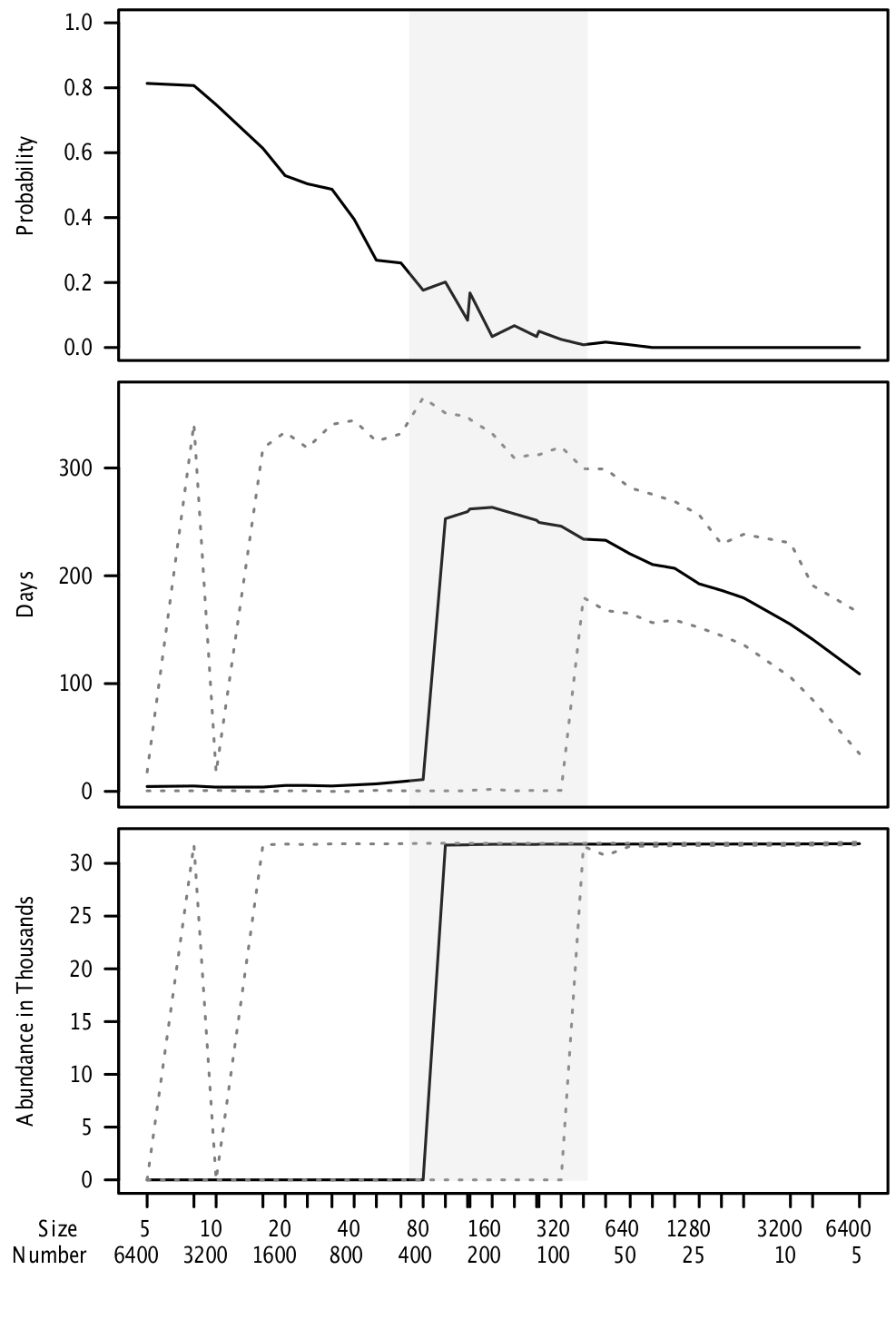

Supplement: Figure S3 — Alternate random network, here a non-directional Barabasi network [48] , with a zero appeal of 1 and a power of preferential attachment of 0.5. For a fixed total single species total population size 32,000, without non-influenza mortality (μ = 0), the effect of changing local patch size and patch number on (A) frequency of epidemic failure, (B) median length of epidemic in days, and (C) median total number of animals infected over 100 simulations. Dotted lines represent the empirical 97.5% and 2.5% percentiles, creating a 95% bootstrap confidence interval. Other parameters are as in Table 1, including environmental transmission, except α = 0.1111, and ω = 0.03, without any infection control program. Gray area represents parameter region where 1< R0<6 within a patch. (TIF) [file pone.0080091.s003.tif]

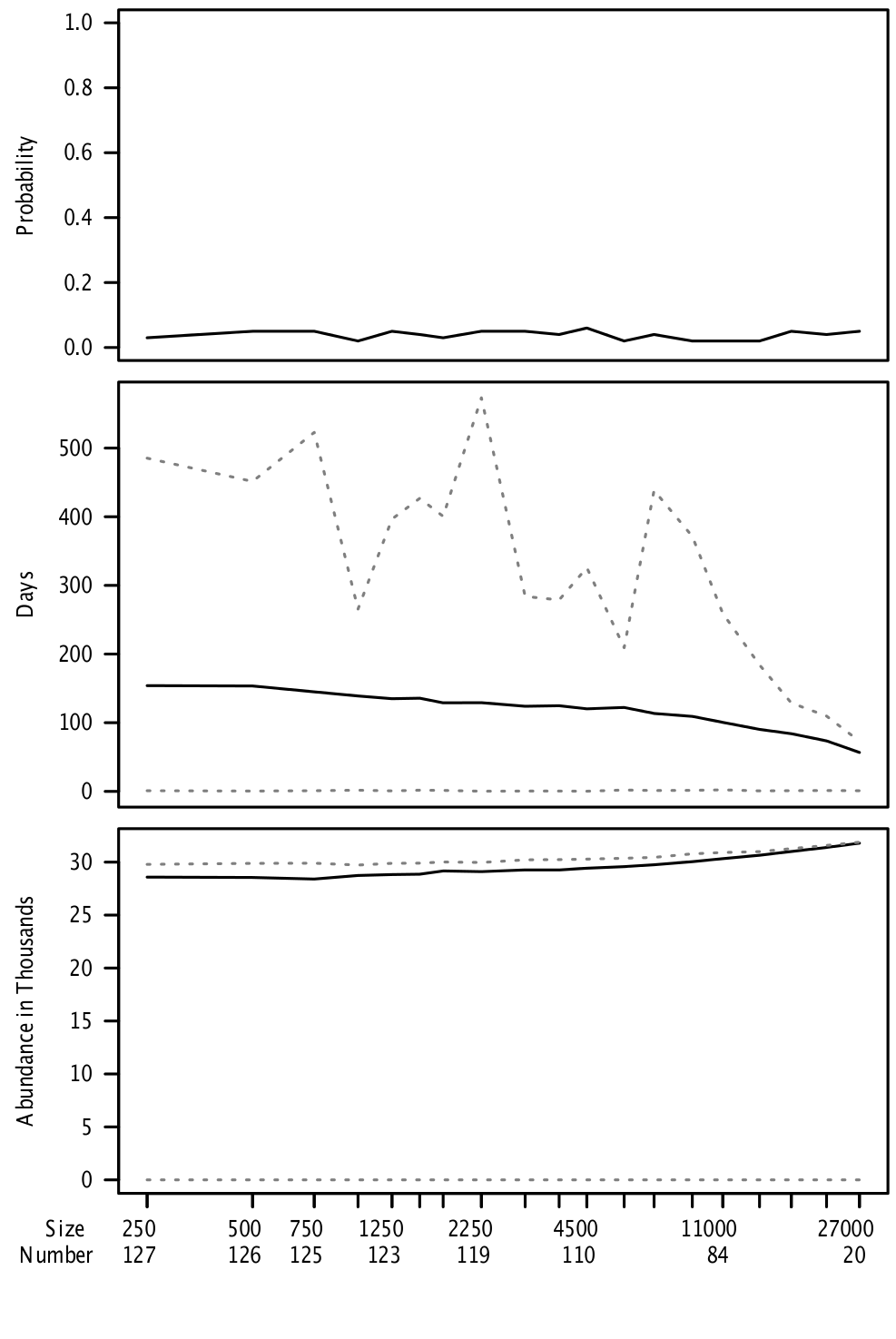

Supplement: Figure S4 — Standard small world network (ρ = 0.6), but there is now one larger farm of variable size. The top row of the x-axis is the size of the larger patch, while the bottom row is the number of smaller patches, all of size 250 hosts. Thus the far left of the graph is the same as for Figure 1 in the main text. For a fixed total single species total population size 32,000, without non-influenza mortality (μ = 0), the effect of changing local patch size and patch number on (A) frequency of epidemic failure, (B) median length of epidemic in days, and (C) median total number of animals infected over 100 simulations. Dotted lines represent the empirical 97.5% and 2.5% percentiles, creating a 95% bootstrap confidence interval. Other parameters are as in Table 1, including environmental transmission, except α = 0.1111, and ω = 0.03, without any infection control program. (TIF) [file pone.0080091.s004.tif]

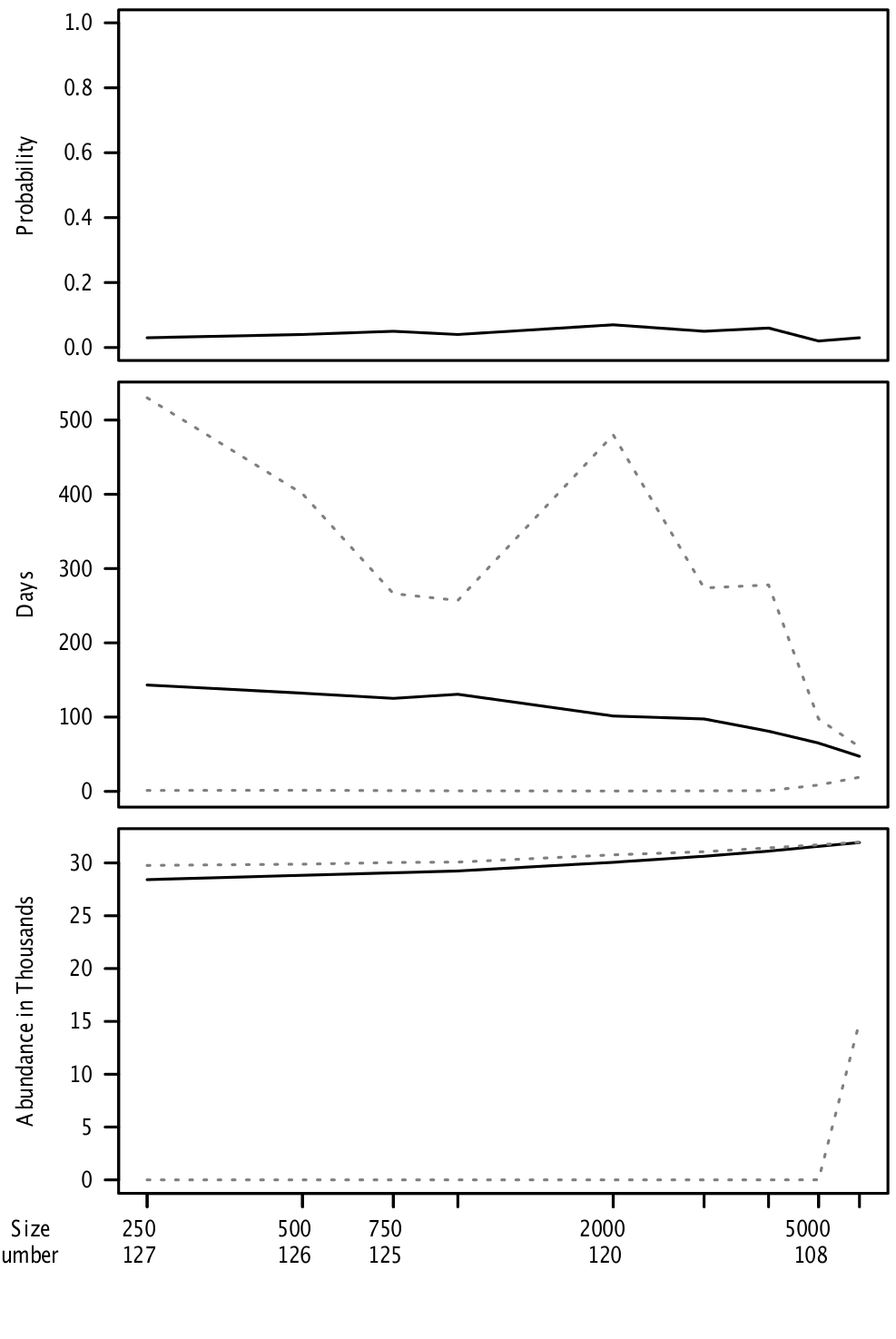

Supplement: Figure S5 — Standard small world network (ρ = 0.6), but there is now five larger farms of variable size. The top row of the x-axis is the size of each of the five larger patches, while the bottom row is the number of smaller patches, all of size 250 hosts. Thus the far left of the graph is the same as for Figure 1 in the main text. For a fixed total single species total population size 32,000, without non-influenza mortality (μ = 0), the effect of changing local patch size and patch number on (A) frequency of epidemic failure, (B) median length of epidemic in days, and (C) median total number of animals infected over 100 simulations. Dotted lines represent the empirical 97.5% and 2.5% percentiles, creating a 95% bootstrap confidence interval. Other parameters are as in Table 1, including environmental transmission, except α = 0.1111, and ω = 0.03, without any infection control program. (TIF) [file pone.0080091.s005.tif]

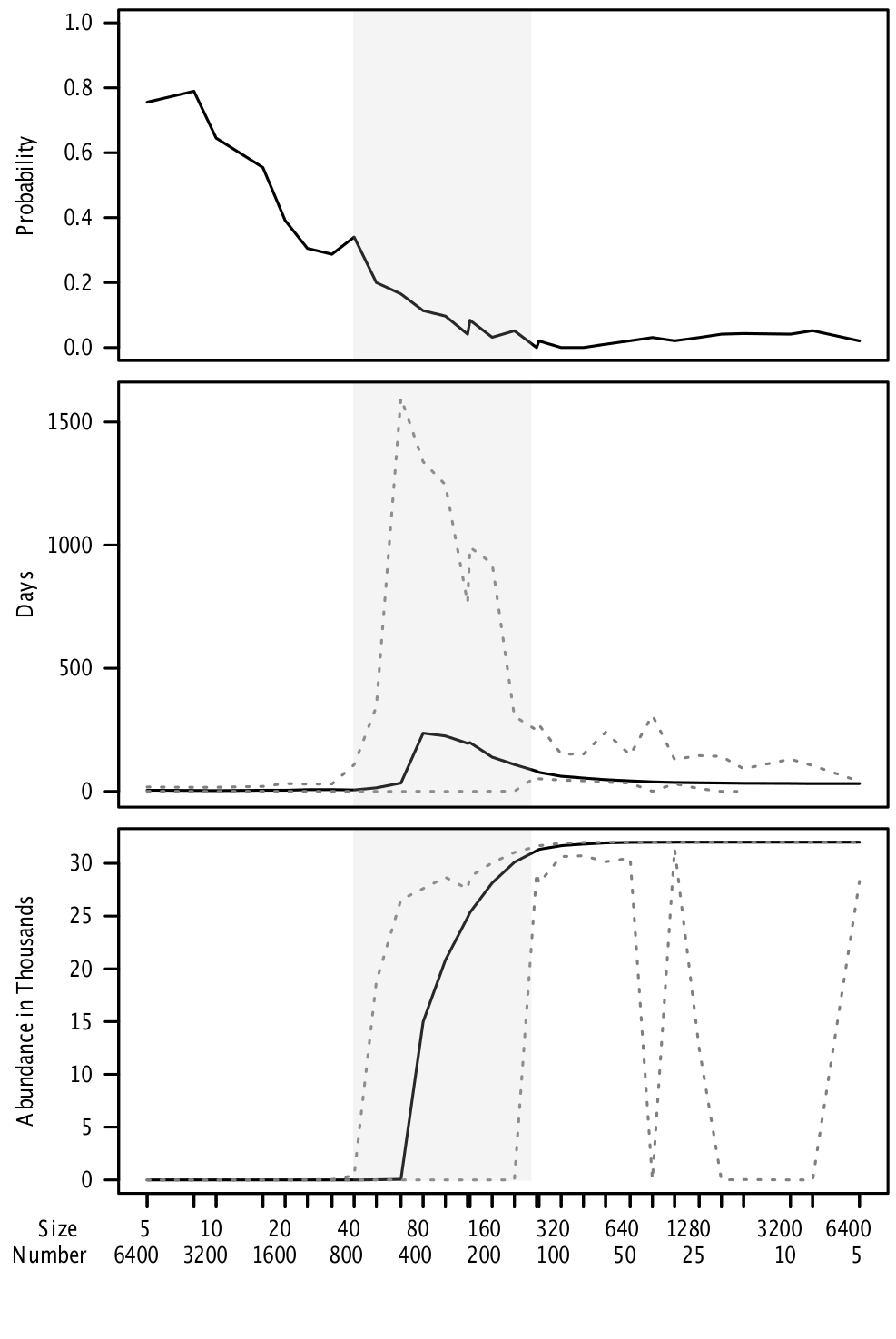

Supplement: Figure S6 — Alternate transmission parameters that more closely resemble H5N1 infections in chickens with no recovery and faster mortality [51] . For a fixed total single species total population size 32,000, without non-influenza mortality (μ = 0), the effect of changing local patch size and patch number on (A) frequency of epidemic failure, (B) median length of epidemic in days, and (C) median total number of animals infected over 100 simulations. Dotted lines represent the empirical 97.5% and 2.5% percentiles, creating a 95% bootstrap confidence interval. Other parameters are as in Table 1, including environmental transmission, except β = 0.0081, γ = 0, α = 0.32, and ω = 0.05, without any infection control program. (TIF) [file pone.0080091.s006.tif]

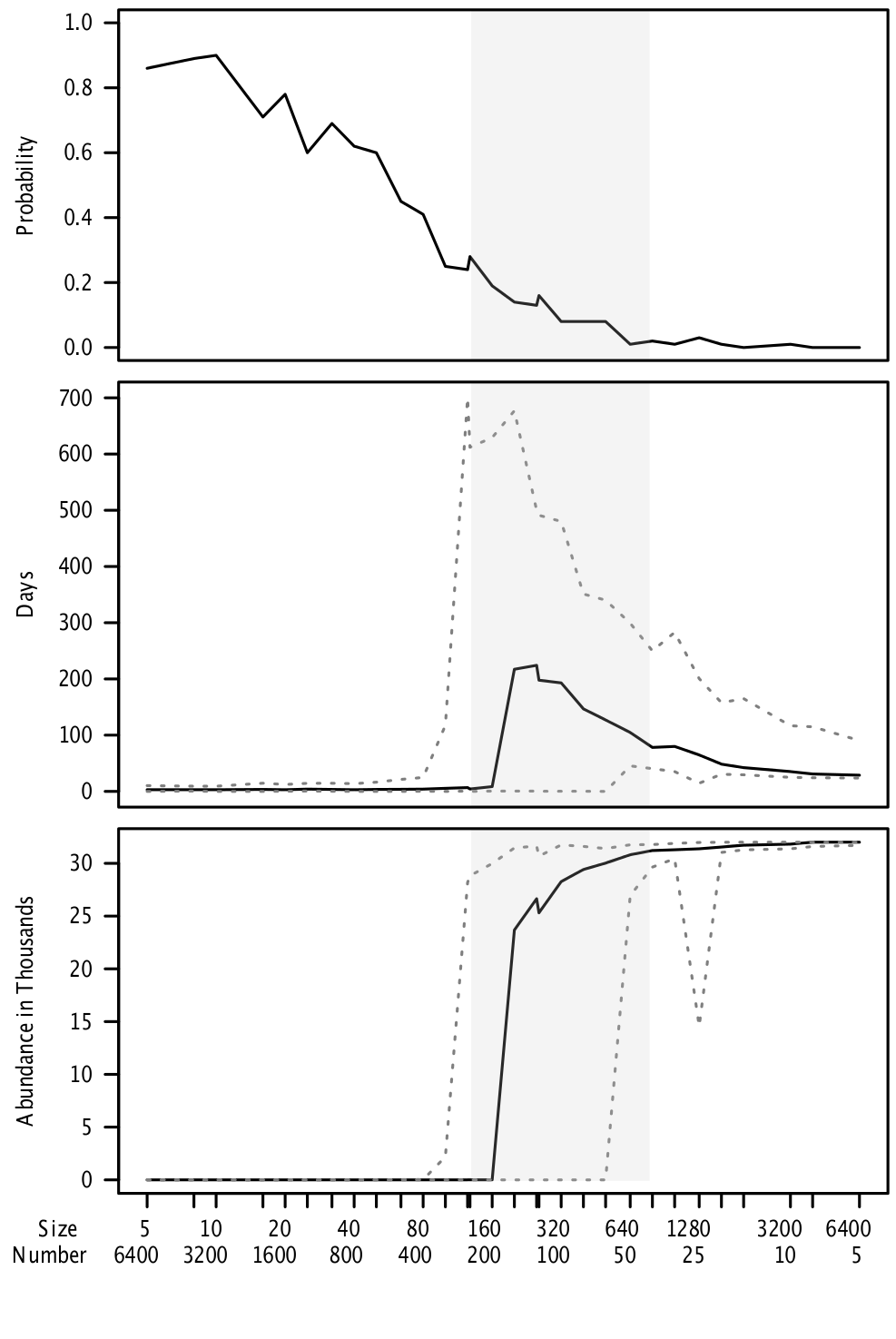

Supplement: Figure S7 — Alternate transmission parameters that emphasize environmental transmission more and direct transmission less, with higher mortality. For a fixed total single species total population size 32,000, without non-influenza mortality (μ = 0), the effect of changing local patch size and patch number on (A) frequency of epidemic failure, (B) median length of epidemic in days, and (C) median total number of animals infected over 100 simulations. Dotted lines represent the empirical 97.5% and 2.5% percentiles, creating a 95% bootstrap confidence interval. Other parameters are as in Table 1, including environmental transmission, except β = 0.003, υ = 0.002, α = 0.222, and ω = 0.143, without any infection control program. (TIF) [file pone.0080091.s007.tif]

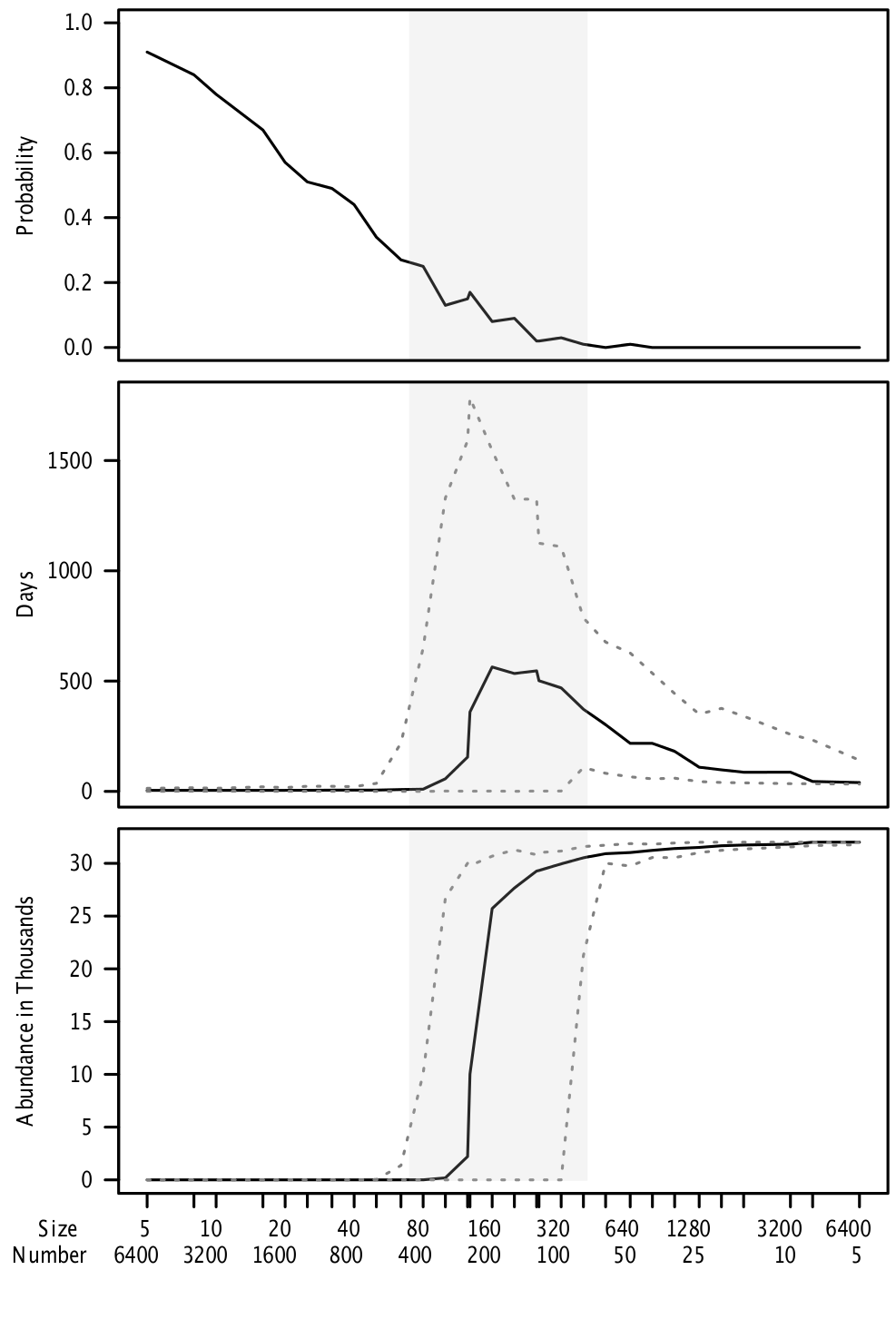

Supplement: Figure S8 — Alternate transmission parameters without environmental transmission. For a fixed total single species total population size 32,000, without non-influenza mortality (μ = 0), the effect of changing local patch size and patch number on (A) frequency of epidemic failure, (B) median length of epidemic in days, and (C) median total number of animals infected over 100 simulations. Dotted lines represent the empirical 97.5% and 2.5% percentiles, creating a 95% bootstrap confidence interval. Other parameters are as in Table 1, excluding environmental transmission, i.e. υ = 0.0, without any infection control program. (TIF) [file pone.0080091.s008.tif]

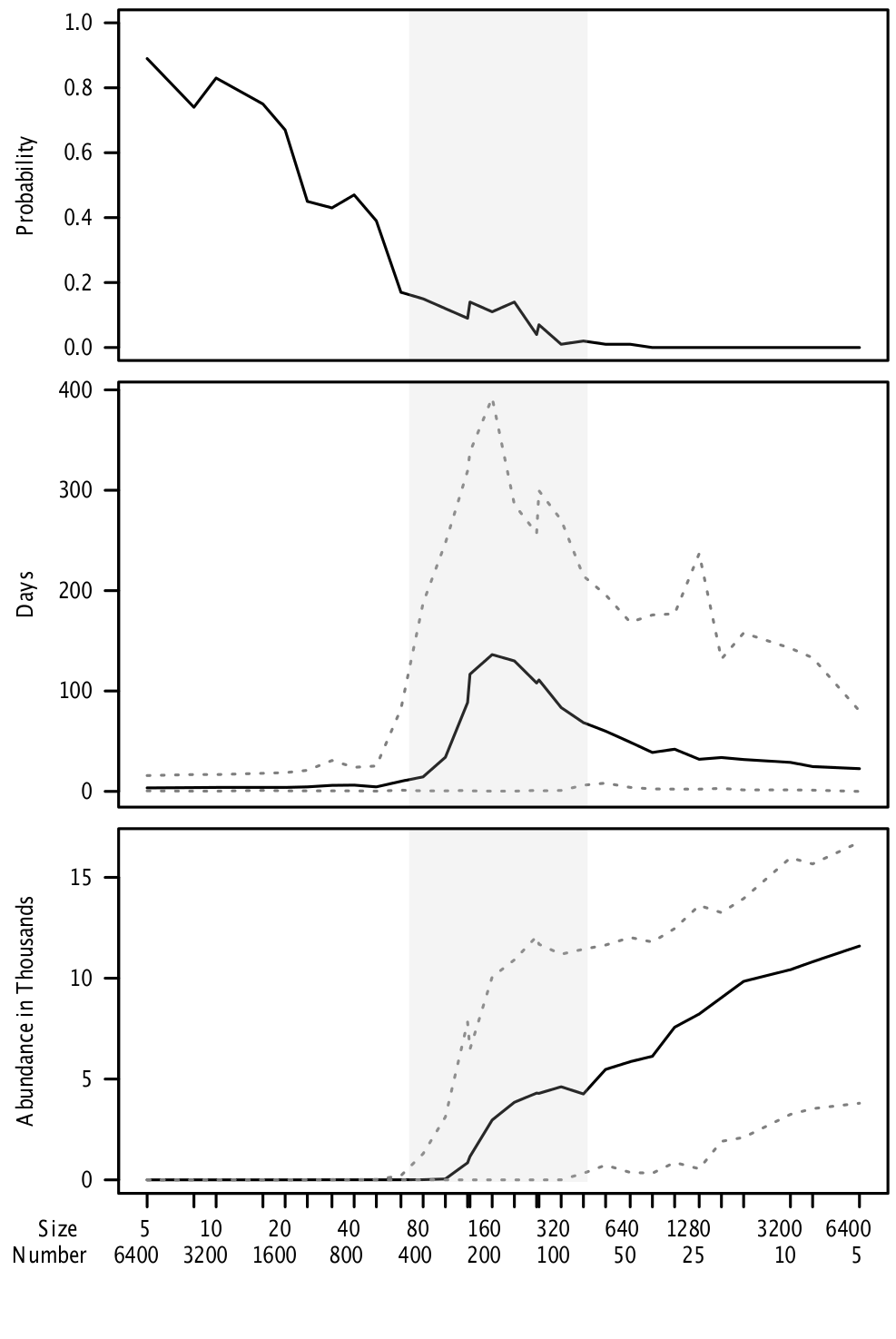

Supplement: Figure S9 — Less effective control program, πReport = 0.1, not 0.9. For a fixed total single species total population size (32,000), without non-influenza mortality (μ = 0), the effect of changing local patch size and patch number on (A) frequency of epidemic failure, (B) median length of epidemic in days, and (C) median total number of animals infected over 100 simulations. Dotted lines represent the empirical 97.5% and 2.5% percentiles, creating a 95% bootstrap confidence interval. Here with control measures implemented, (πReport = 0.1, πDetect = 0.9, τCrit = 1, ICrit = 5). (TIF) [file pone.0080091.s009.tif]
